# Development, implementation and evaluation of a digital treatment for adolescents with chronic pain: a protocol for a multi-phase study

**DOI:** 10.3389/fdgth.2025.1555733

**Published:** 2025-06-04

**Authors:** Jordi Miró, Ariadna Sampietro, Sonia Monterde, Pablo Ingelmo, Rikard K. Wicksell, Carme Nolla, Mercedes Alonso, Juan José Lázaro, Ernesto Martínez, Paloma Rubio, Armando Sánchez, Vanessa Sánchez, Alvaro Vázquez, Rocío de la Vega, Francisco Reinoso-Barbero

**Affiliations:** 1 Unit for the Study and Treatment of Pain—ALGOS, Research Center for Behavior Assessment (CRAMC), Department of Psychology, Universitat Rovira I Virgili, Tarragona, Spain; 2 Institut Sanitària Pere Virgili, Universitat Rovira I Virgili, Tarragona, Spain; 3 Unit of Physical Therapy, Department of Medicine and Surgery, Faculty of Medicine and Health Sciences, Universitat Rovira I Virgili, Tarragona, Spain; 4 Department of Anesthesiology, Montreal Children’s Hospital, The Edwards Family Interdisciplinary Centre for Complex Pain, Montreal, QC, Canada; 5 Department of Anesthesia, McGill University, Montreal, QC, Canada; 6 Research Institute, Alan Edwards Centre for Research on Pain, McGill University Health Center, Montreal, QC, Canada; 7 Department of Clinical Neuroscience, Karolinska Institutet, Stockholm, Sweden; 8 Pain Clinic, Capio St Göran Hospital, Stockholm, Sweden; 9 Health Care Center Xarxa Tecla, Tarragona, Spain; 10 Pediatric Pain Unit, Pediatric Anesthesiology Department, La Paz University Hospital, Madrid, Spain; 11 Department of Anaesthesia and Pain Therap, Hospital Sant Joan de Déu, Barcelona, Spain; 12 Department of Anesthesia, Children University Hospital Niño Jesús, Madrid, Spain; 13 Pediatric Chronic Pain Unit, Anesthesia Department, 12 de Octubre Universitary Hospital, Madrid, Spain; 14 Pediatric Pain Unit, Pediatric Anaesthesiology Section, Critical Care and Pain Therapy Department, Miguel Servet University Hospital, Zaragoza, Spain; 15 Servei d’Anestesiologia, Reanimació I Tractament del Dolor, Hospital Infantil I Hospital de la Dona, Hospital Universitari Vall d’Hebron, Barcelona, Spain; 16 Servicio de Anestesiología y Reanimación, Unidad de Dolor, Hospital Universitari Son Espases, Palma, Spain; 17 Department of Personality, Evaluation, and Psychological Treatment, Faculty of Psychology and Speech Therapy, University of Málaga, Málaga, Spain; 18 Instituto de Investigación Biomédica de Málaga y Plataforma en Nanomedicina (IBIMA Plataforma BIONAND), Málaga, Spain

**Keywords:** adolescents, chronic pain, digital health, digital therapeutics, mobile application, pain management

## Abstract

Chronic pain in adolescents is an increasing public health concern with a significant physical, psychological, and social impact. This study aims to develop, implement, and evaluate DigiDOL-Ad, a digital psychosocial treatment for adolescents with chronic pain, supplemented by dedicated websites for their parents and teachers. This multicenter study will be conducted in four phases: (1) Development of the intervention framework and foundational planning; (2) Focus groups with adolescents with chronic pain, their parents, teachers, healthcare professionals and health authorities to identify specific needs and tailor the psychosocial treatment and related components; (3) Iterative usability testing of the digital treatment, using an a hermeneutical circle methodology to refine the design based on participant feedback; and (4) Evaluation of DigiDOL-Ad through a pre-treatment, post-treatment, and 3-month follow-up assessment. DigiDOL-Ad has the potential to improve the quality of life for adolescents with chronic pain. By leveraging digital health technologies, this innovative approach could establish a new benchmark for treating adolescents with chronic pain, emphasizing interdisciplinary and stakeholder-driven care.

**Clinical Trial Registration:**

clinicaltrials.gov, identifier NCT06765200.

## 1 Introduction

Chronic pain in adolescents is a significant and growing concern. Research has shown that the prevalence of chronic pain in adolescents is high and increasing (1, 2), including the most severe cases, that is, those with the highest levels of pain intensity and interference (3). A recent review reported that prevalence rates vary depending the study, however, it is estimated that about 25% of adolescents report chronic pain (4). This condition leads to substantial impairments in physical (e.g., sleep) and psychological (e.g., depression) well-being (5, 6), affecting their cognitive function (7) as well as school performance (8). Moreover, it also interferes with their social function (9, 10).

There is widespread consensus that a psychosocial approach is essential for maintaining or improving the functioning and well-being of adolescents with chronic pain (11, 12). However, the availability of evidence-based psychosocial treatments for adolescents with chronic pain is insufficient to meet the high demand, particularly in areas outside large cities. The reasons vary, but one of the main barriers is the shortage of professionals trained to attend the demands (13). As a result, many adolescents endure chronic pain without the necessary support for satisfactory daily functioning and quality of life, highlighting a severe inequality in access to adequate care (14). Due to this shortage of treatment programs, many adolescents with chronic pain are attended first by primary care physicians (15). These physicians, however, typically refer patients to specialized programs, leading to unnecessary delays as patients face long waitlists to receive proper specialized care (16, 17).

Digital health, defined as “the field of knowledge and practice associated with the development and use of digital technologies to improve health” (18), provides new opportunities to increase the reach of evidence-based psychosocial interventions for chronic conditions, including chronic pain. Digitally delivered psychosocial interventions have shown similar effects to standard (face-to-face) treatment for conditions such as anxiety and depression (19), and the empirical support for digital health in the field of chronic pain is rapidly increasing (20, 21). Digitalization represents a paradigm shift in healthcare with dramatically improved opportunities to increase patient access, equality, and equity (22). However, evidence-based digital health treatments are not available for chronic pain patients in regular care, including adolescents, outside clinical trials (23, 24).

### 1.1 Objectives

The overarching objective of this project is to help reduce the pain impact in adolescents with chronic pain and improve their functioning. We aim to achieve this by developing and implementing DigiDOL-Ad, a novel and reliable digital treatment (i.e., mobile application). With DigiDOL-Ad we aim to empower patients to better manage and cope with their chronic pain. In addition, this new app will be complemented by websites with resources and information, one specifically developed for parents or main caregivers and another for teachers. This article describes the protocol created for this project and follows the Standard Protocol Items: Recommendations For Interventional Trials (SPIRIT) guidelines (25) (see Supplementary Appendix 1 for the WHO Trial Registration Data Set information).

In pursuit of this goal, the project will evolve with specific objectives:

1. Improve the understanding of the needs of all stakeholders—adolescents with chronic pain, their parents/tutors, attending physicians, teachers, and health authorities—regarding the management of chronic pain and the features of DigiDOL-Ad. We expect to find many important, specific to each group, and shared, unmet needs (Hypothesis 1).
2. Co-create a scientifically validated and evidence-based, patient-centered digital treatment for adolescents with chronic pain, involving all stakeholders: adolescents with chronic pain, parents, attending physicians, teachers, and health authorities. We expect that the digital treatment will be found user-friendly, and liked by all participants (Hypothesis 2).
3. Evaluate DigiDOL-Ad in helping adolescents adjust to and manage chronic pain. We anticipate that adolescents with chronic pain using DigiDol-Ad will report statistically significant improvements in chronic pain and physical and psychosocial functioning after treatment, maintained at follow-up (Hypothesis 3).
4. Assess whether the active involvement of parents and teachers, facilitated through dedicated companion websites, as change agents, that contribute to improved outcomes in adolescents with chronic pain. We hypothesize that adolescents whose parents and teachers actively engage with the digital treatment will experience greater improvements in pain management and quality of life compared to those with less engaged support (Hypothesis 4).

## 2 Materials and methods

### 2.1 Study design

**Figure 1:**
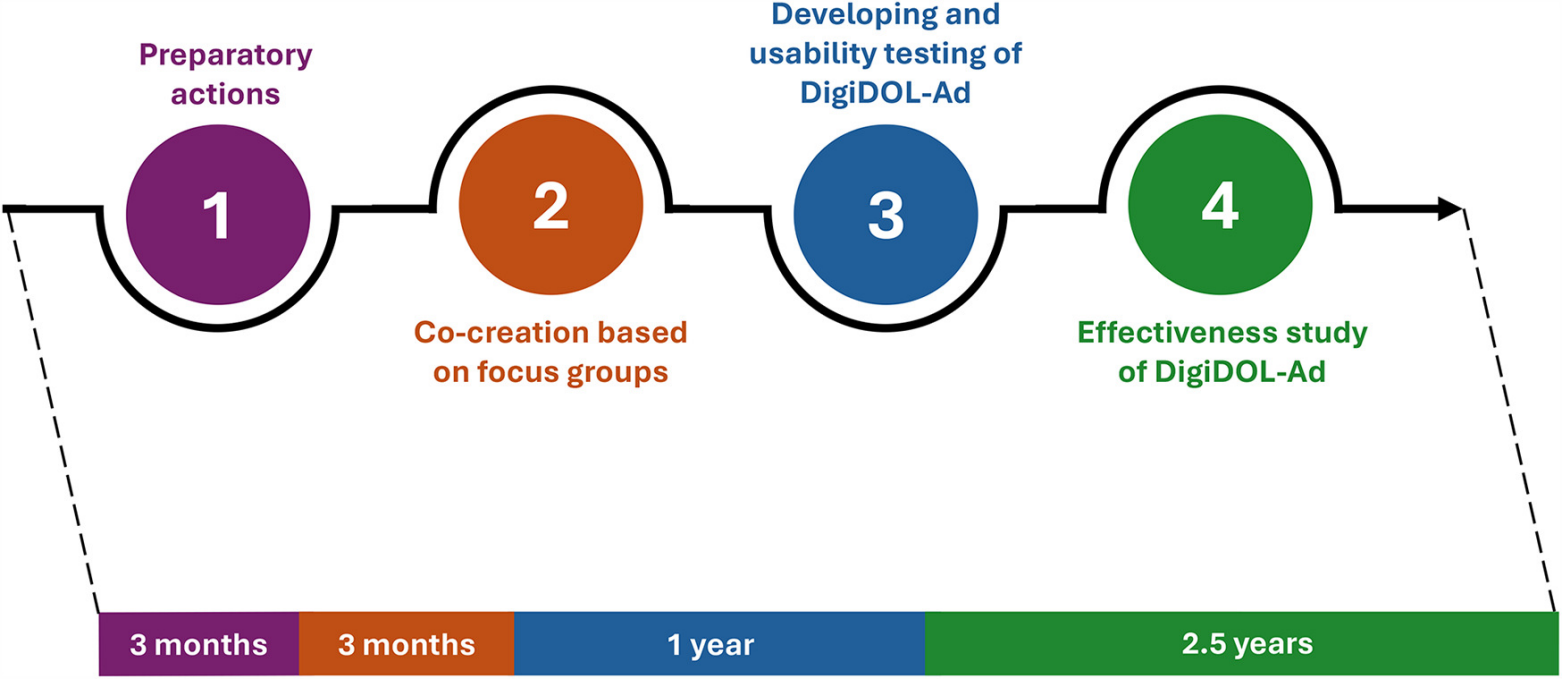
Summary of study procedures.

This is a study involving 4 phases with specific procedures (see Figure 1). The study design follows the Medical Research Council (MRC) Framework for developing and evaluating complex interventions (26).

### 2.2 Procedure: description of the study's phases

#### 2.2.1 Phase 1. Preparation: laying the groundwork for future studies

This phase is critical to establishing a solid foundation for the app's development and ensuring the quality of subsequent studies. The preparatory actions involve several key steps, focusing on collaboration, logistical planning, and resource alignment, including: (a) establishing partnerships with healthcare professionals (i.e., attending physicians) and hospitals (i.e., scheduling meetings to explain the project objectives and gather insights; partnering with hospitals to act as research sites and recruitment hubs for study participants); (b) obtaining ethical and logistical approvals to ensure that the project complies with ethical standards and is logistically viable (i.e., submitting the project proposal to institutional review boards and ethics committees for approval; developing consent and assent materials tailored to adolescents and their guardians); (c) developing study protocols to guide empirical studies and ensure consistency in data collection (i.e., defining participant inclusion and exclusion criteria; drafting standardized procedures for introducing the app to potential participants, including how instructions will be provided and how data will be collected; developing contingency plans to address potential challenges, such as dropout or technical issues); and (d) process pilot testing to refine protocols and ensure all systems are functioning optimally before full-scale implementation (i.e., testing recruitment processes, consent procedures, and data collection tools; making necessary adjustments to the protocols and app design based on feedback from pilot phase).

#### 2.2.2 Phase 2. Co-creation of DigiDOL-Ad

This phase focuses on identifying the needs of all stakeholders. We will develop the DigiDOL-Ad with co-creation and user involvement at all phases to identify real user needs and design the treatment to meet these needs. The co-creation process for the mobile app will leverage insights from focus groups (FGs) to ensure the app is user-centered, inclusive, and tailored to meet the needs of adolescents with chronic pain and all stakeholders involved in managing chronic pain. The protocol for this phase will address participant diversity (i.e., FGs will be gender balanced, and include participants from different backgrounds and varying levels of digital literacy) and integration of feedback into the app development process to design an app that is practical, engaging, and effective.

Each FG will take between 60 and 90 min. In these sessions, participants will be asked to report about: (a) current challenges and unmet needs in relation to managing pain, (b) desired features and design (interface simplicity, visual style), functionalities, preferences about the content, activities to be included in the mobile app and barriers to adoption and usage of the app. Adolescents and their parents will also share their experiences with the treatments received in the past, including the pros and cons related to the treatments received, and their preferences regarding the use of apps (and websites).

Additionally, we will conduct FGs with teachers to understand the needs within the school context (i.e., they will be asked about their knowledge of chronic pain, their experiences with students suffering with chronic pain, the needs they identify in the school context and the strategies and resources they consider useful) and develop the web page [i.e., we will gather their opinions and preferences about the (co)creation of a website to support students with chronic pain].

Moreover, we will also conduct FGs with healthcare professionals to discuss the treatments they provide, barriers to treatment, their experiences with individuals with chronic pain, and their experience with and position around the use of digital treatment in healthcare practice.

Finally, we will conduct a FG with health authorities to determine the key characteristics that would enhance DigiDOL-Ad's appeal for integration into the mainstream healthcare system and to identify strategies for simplifying and streamlining this process. All FGs will be audio recorded and transcribed verbatim. In addition, we will also conduct interviews at the end of each FG to ensure that did not miss any significant bit of information. Following the recommendation of Braun and Clarke (27), we will conduct focus groups until reaching the point of saturation. This refers to the stage where no new themes or codes emerge from the data (i.e., information redundancy). At this stage of the research, it is not possible to determine this point entirely in advance.

#### 2.2.3 Phase 3. Development and usability testing of DigiDOL-Ad and accompanying websites

In this phase, DigiDOL-Ad and the accompanying websites will be developed. Psychologists with extensive experience in the treatment of adolescents with chronic pain, a software engineer and a graphic designer, all with wide experience in the development of health applications [e.g., (23, 28–30)], will develop DigiDOL-Ad and the websites, leveraging the data obtained in earlier phases. In this process, we will use the *mHealth assessment and development guideline* [MAG; (24, 31, 32)] to help guide the development and ensure that DigiDOL-Ad meets the expected safety, security, privacy, usability, transparency, appropriateness and usability requirements for a health app. This treatment will have a psychosocial focus. Drawing from our prior development of digital treatments [e.g., (30)], we anticipate that the duration of the current intervention will be approximately 8 weeks. Importantly, DigiDOL-Ad will be designed to be a stand-alone intervention. That is, adolescents with chronic pain will not have the option to interact with a therapist (or with other adolescents) and the treatment itself will be self-guided as we have already implemented in previous apps with significant positive results (23, 28, 30). The digital treatment will be developed on data-driven clinical guidelines, such as World Health Organization's guidelines (12) on the management of chronic pain in children. The application will be developed for both the Android and iOS platforms to ensure widespread accessibility. Furthermore, its potential integration into the Spanish healthcare system will be explored.

A website for parents and a website for teachers will be also developed as companions to DigiDOL-Ad. The objective is that both parents and teachers act as active change agents to help adolescents with chronic pain improve. The content of the parents' web page will be based on the adolescent's treatment and will provide suggestions on what to do and not do to support adolescents in progressing though the treatment. Given that adolescents spend a significant portion of their time at school and the critical role it plays in their overall development, we will also develop a website for teachers. This website will provide resources and information to support students with chronic pain. The content will be based on the information gathered with the FGs with teachers and will be aimed at providing practical resources, strategies, and information tailored to the school setting. The general idea is that the content should empower teachers to understand, accommodate, and assist students while fostering an inclusive and supportive learning environment.

The usability study of DigiDOL-Ad will be conducted with groups of adolescents with chronic pain and is based on the concept of a “hermeneutical circle”. This involves an iterative process of implementing a design, gathering insights through discussions and feedback, and refining the design in successive cycles until it becomes user-friendly and minimizes errors. Based on previous experiences (23, 33), we expect that 2–3 cycles will be required, with each session taking about 45 min. Participants in these tests will be informed of the purpose, procedures, risks, and asked to sign an informed consent or assent to participate (see Supplementary Appendix 2 for a copy of the consent/assent forms to be used in this study). Participants will engage with DigiDOL-Ad using only the standardized instructions embedded within the app, as it is designed to be self-explanatory and guide users independently through the content. A “concurrent thinking” protocol will be employed, during which participants' actions will be observed, and detailed field notes will be recorded and transcribed. Errors made during app usage will also be documented. Following the completion of the assigned tasks, participants will respond to open-ended questions regarding the app's usability, efficiency, and overall satisfaction, based on methodologies established in prior comparable studies (30).

#### 2.2.4 Phase 4. Evaluation study of DigiDOL-Ad

In this last phase, we will evaluate DigiDOL-Ad. This will be a multi-site study with 8 hospitals referring potential participants. The procedure will begin with adolescents and their parents responding to a battery of questionnaires. This assessment will be conducted online, to decrease biases due to social desirability effects. We will use the *REDCap System* (34) to implement the online assessment, which allows for programing follow-ups at the different data collection points. Participants will be assessed three times: Pre- and Post-treatment (T1, T2) and 3-months follow-up (T3, adolescents only). After completing T1 assessment, participants will receive access to DigiDOL-Ad (adolescents) and to the webs (parents and teachers).

Participants will receive written instructions detailing how to access the treatment. Each participant will be assigned a unique username and password, which will be linked to their participant ID. This linkage will facilitate the monitoring of participants' adherence.

To maximize retention of participants the project will simplify accessibility and provide easy-to-understand instructions for downloading, accessing, and using the app. In addition, reminder emails with personalized and motivational messages will be sent at each assessment point. Moreover, we will conduct periodic surveys or check-ins every week to identify barriers to participation, such as time constraints or usability issues, and participants will receive compensation for completing the surveys. In addition, to maximize engagement from the healthcare professionals, they will receive information on the study progress and preliminary findings -when possible- using periodic newsletters to sustain interest. Moreover, they will be informed about the referral and enrolment volumes per hospital, emphasizing the value of their contribution to the study and its potential impact on improving chronic pain management in adolescents. Importantly, the project will acknowledge participants' efforts through individual thank-you notes and public acknowledgment in study updates. Finally, the project will build trust and rapport by facilitating transparent communication with a study coordinator to build a personal connection with participants.

In the check-ins mentioned earlier, open-ended questions will be included, to allow participants to report any unintended effects of the intervention and identify potential causes. This feedback will facilitate necessary improvements to the intervention. All collected information will be securely stored on the same servers as the other study data.

To ensure transparency and compliance, any important protocol modifications in the research project will be communicated promptly and appropriately to all relevant parties. Our plan outlines the steps, responsibilities, and channels for effectively disseminating such changes to: (a) research team members (i.e., immediate notification followed by detailed discussions in the next scheduled meeting and revised protocol documents shared via secure project management platforms); (b) research ethical committee (i.e., formal submission of an amended protocol along with supporting documentation including a rationale for changes and a complete risk assessment); (c) participants (i.e., direct communication via email, app notifications, or phone calls, depending on participant's preference); (d) trial registry (i.e., update clinical trial registry records to reflect modifications). Moreover, we will maintain a log of protocol modifications, communication records, and acknowledgments from all parties, and store updated documents in a centralized and secure location accessible to the research team.

We plan to disseminate the project's results, targeting both general audiences and specialized groups. This will include social media and communication through patient associations. Likewise, we will share the findings in scientific forums like specialized scientific journals, meetings and congresses. Additionally, we will send email updates to all the stakeholders that participated to ensure transparency and broad accessibility to the outcomes.

### 2.3 Participants

For adolescents interested in participating, they must meet the following *inclusion* criteria: (1) being between 12 and 18 years of age; (2) having a non-oncology secondary chronic pain problem (as defined by ICD-11 (35); (3) having internet access; (4) having an iOS- or Android-based mobile phone; (5) a parent willing to participate in the study; and (6) providing informed consent/assent. Potential participants will be *excluded* if they have cognitive or language problems that could interfere with the correct understanding of the procedure and the questionnaires that will be used. Participants will not be restricted from engaging in concomitant interventions.

In the *usability study*, as recommended (28), participants will include 5 end users (i.e., adolescents with chronic pain) per cycle, and 2 or 3 cycles are expected (*N* = 15).

In the evaluation study, the recruitment of participants will take place at the 8 following Spanish hospitals: Hospital Universitario Infantil Niño Jesus, Hospital Sant Joan de Déu, Hospital Universitario La Paz, Hospital de Sant Pau i Santa Tecla, Hospital Universitario Miguel Servet, Hospital Universitari Son Espases, Hospital Universitari Vall d'Hebron, and Hospital Universitario 12 de Octubre. Hospital representatives will distribute informational brochures to adolescents and their families, outlining the details of the project and providing our contact information. This ensures that those interested can easily initiate direct communication with the research team.

Referred adolescents and their parents will sign informed consents or assents, as appropriate, if interested in participating. This involves providing a document describing in detail the study procedures and risks to interested adolescents and parents. This procedure has been described in the documents provided to the Human Subjects Ethics Committee of the Universitat Rovira i Virgili and approved by that Committee (CEIPSA-2023-PR-0033). If the adolescent and parent are willing to participate, they will sign the electronic informed consent/assent document prior to any study-related activity (36). The document will then be received by the Principal Investigator and authorized members of the research team.

In this study, the sample will include adolescents with chronic pain and one of their parents. Based on a within-subjects repeated measures ANOVA with three time points (baseline, post-treatment, and 3-month follow-up), we will use GPower (version 3.1.9.7) to calculate the required sample size. We set a small effect size (f = 0.10), with a two-tailed alpha of 0.05 and power of 0.80. For the calculation, the following parameters were assumed: number of measurements = 3 (baseline, post-treatment, and 3-month follow-up), correlation among repeated measures = 0.5, and nonsphericity correction epsilon = 1. This calculation indicated that a sample of *N* = 163 dyads would be sufficient. However, in order to prevent attrition issues, we will be recruiting a minimum of 195 dyads -minimal attrition is expected based on prior studies on digital treatments with similar populations (37). Recruitment is expected to proceed at a rate of approximately 5 participants per month, based on projections from similar previous studies [e.g., (30)]. To account for potential variability, we conservatively estimate a recruitment rate of 2 adolescents per month per hospital. With 8 hospitals participating, this would result in enrolling the required sample of 352 participants within 22 months. An additional 5 months will be allocated for participants to complete the treatment (approximately 8 weeks, as previously described) and the 3-month follow-up, bringing the total duration for recruitment, enrollment, and follow-up to 27 months.

In order to ensure that the target sample size is reached in the evaluation study, the following strategies will be implemented: (a) using clear and engaging messages (i.e., developing age-appropriate, visually appealing recruitment materials, like posters, flyers, or videos to be shared by hospitals and healthcare professionals); and (b) highlighting the benefits of participation, such as improving their own pain management and contributing to advancements in adolescent healthcare. Moreover, we will offer both monetary and non-monetary incentives (i.e., certificates of participation and feedback on their progress). To enhance participant retention and ensure completion of follow-ups, we plan to implement personalized reminders. These reminders will be delivered via app notifications, SMS, or email, and will serve to notify participants of upcoming tasks or scheduled follow-up activities.

We also plan to collect key outcome data from participants who discontinue or deviate from the intervention protocol to minimize data loss and reduce bias, both using direct (i.e., exit survey and interviews to capture feedback and reasons for withdrawal) and indirect or passive data collection procedures (i.e., using app analytics to record usage patterns and adherence metrics prior to discontinuation).

In this project, all participants will be compensated for their participation.

### 2.4 Outcomes and measures

The outcomes used are based on the recommendations of a recent consensus study involving several stakeholders [children with chronic pain, parents, healthcare experts and researchers; (38)]. The main outcomes of the evaluation study are pain intensity, pain interference and the global impression of change after treatment. In addition, we will collect information from a group of secondary outcomes. To capture the overall impact of the intervention, we will assess global impression of change using a standardized measure as suggested in the Core Outcomes in Pediatric Persistent Pain Workgroup (39).

Both adolescents and their parents will respond to an online survey including questions about sociodemographic characteristics, concomitant treatments and pain medication (type and doses). In addition, adolescents will complete the following outcome measures: pain intensity [NRS-11; (40)], pain interference [PROMIS Pediatric Pain Interference; (41)], pain-related self-efficacy [PSEQ; (42)], concerns about pain [UW-CAP; (43)], pain attitudes [Peds-SOPA; (44)], functional disability [FDI; (45)], anxiety [PROMIS Pediatric Anxiety; (46)], depressive symptoms [PROMIS Pediatric Depression; (46)], sleep disturbance [PROMIS Pediatric Sleep disturbance; (47)], fatigue [SFS; (48)], treatment expectancies (49), treatment perceptions [perception of how easy to use and how helpful the skills taught are; (50)], adherence (i.e., use of DigiDOL-Ad and of the skills learned with treatment), global impression of change after treatment (38), satisfaction with treatment (51) and adverse effects (52).

**Figure 2:**
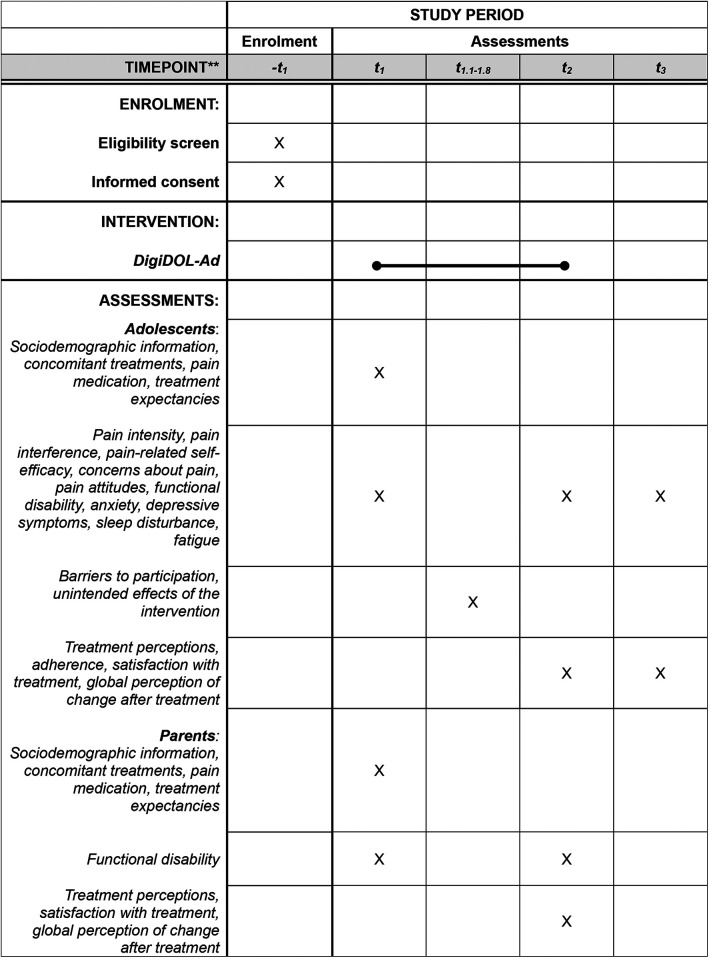
Schedule of enrolment, intervention and assessments for phase 4. t_1_ = enrollment; t_1_ = pre-treatment assessment_;_ t_1.1−1.8_ = weekly check-ins; t_2_ = post-treatment assessment; t_3_ = 3-month follow-up assessment.

In addition, the parents' online survey will include the following outcome measures: functional disability [FDI; (45)], treatment expectancies (49), satisfaction with treatment (51), and global perception of change after treatment (38). Figure 2 provides details of the SPIRIT schedule of enrolment, intervention and assessments.

Our data management plan prioritizes the privacy and anonymity of participants. To ensure anonymity, participants will be identified in evaluation questionnaires and data analyses using a numerical code. Specialized data protection measures, including password protection, will be implemented as needed to safeguard sensitive information. All collected data will be stored in a password-protected database, accessible exclusively to authorized members of the research team. Data will be processed in aggregate form and used solely for scientific research purposes.

**Figure 3:**
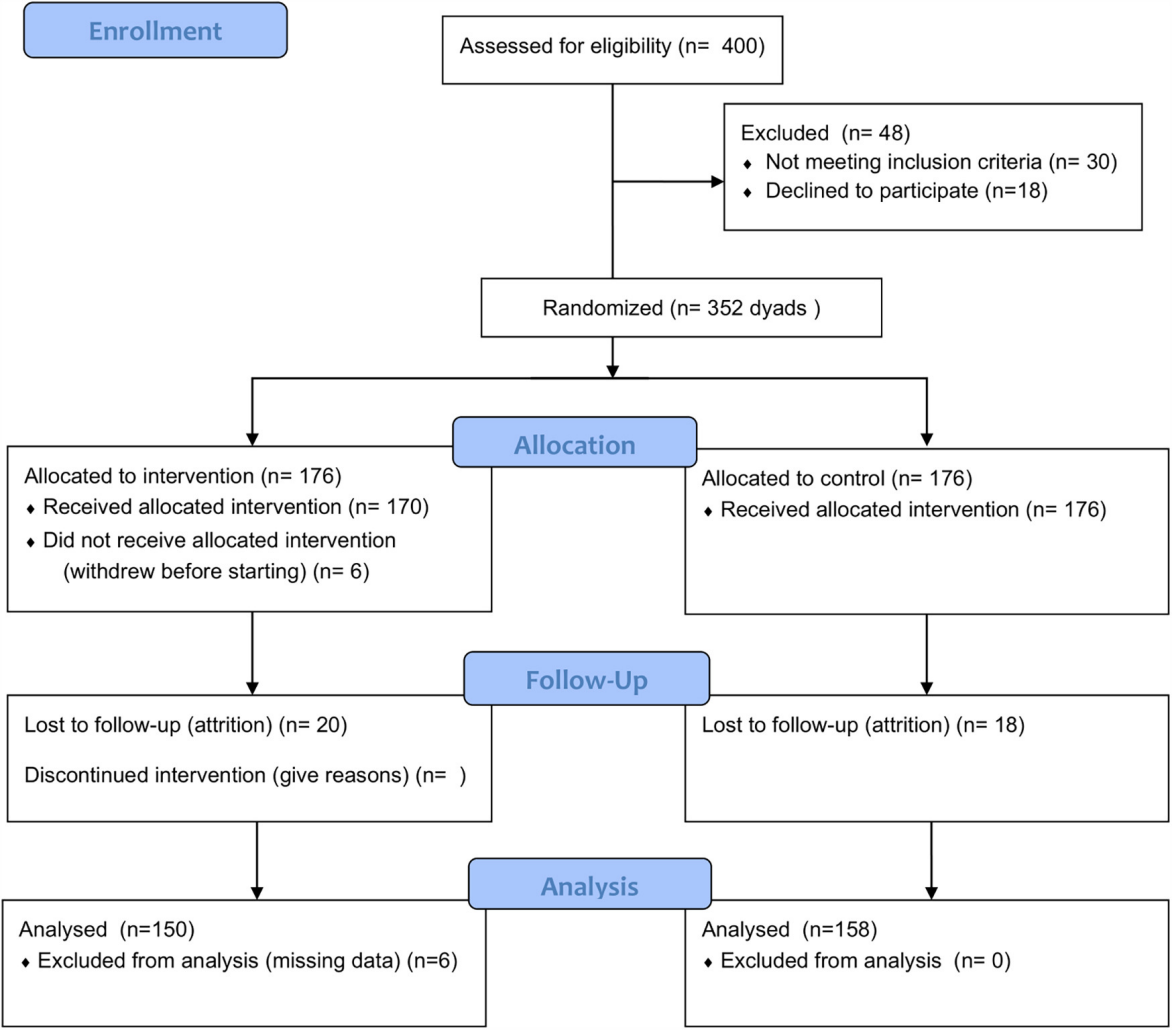
Consort diagram.

Data will be collected using the platform REDCap and stored in secure servers located at University Rovira i Virgili. Only the Principal Investigator will have access to the data, and he will give access to study personnel, when appropriate for analysis and report writing. Once the study is finished, anonymized data will be available at the repository CORA.RDR, which is an online platform designed for storing and sharing research data supported by the Department of Research and Universities of the Government of Catalonia. Figure 3 provides a Consort diagram (the numbers presented are based on predictive estimates derived from our initial enrollment and attrition forecasts. We have made every effort to ensure these figures are as realistic as possible, but they remain projections and may change once actual recruitment and follow-up data become available).

### 2.5 Data analysis

We will use descriptive statistics to describe the sample (i.e., means, standard deviations, and percentages). To study the outcomes of the FGs, we will use a deductive content approach to synthetize the information. After transcribing the sessions, they will be coded, and results extracted and summarized. To study intervention effects, we will perform repeated measures ANOVAs, as described in section 2.3. Effect sizes will be estimated using partial eta squared (*η*ₚ²) for the repeated measures ANOVA, which is recommended for within-subjects designs. In cases where pairwise comparisons are conducted, Cohen's d will be reported to facilitate interpretation of the magnitude of differences. The analysis will follow and intent-to-treat approach, including all participants who initiated the digital treatment, regardless of their level of adherence to the protocol. For handling missing data, multiple imputation methods will be employed to account for potential biases and ensure the robustness of the results. The software IBM SPSS Statistics for Windows will be used.

## 3 Discussion

### 3.1 Study significance

The overarching objective of this project is to help reduce pain impact and improve funtioning in adolescents with chronic pain by empowering them (and their families) to better adjust and cope with chronic pain related problems thorough the digital treatment that we will develop.

One of the major challenges facing the health system is the increasing demand from individuals with non-communicable chronic diseases. Research indicates a significant rise in the prevalence of chronic pain among adolescents (2). Moreover, several of the factors that are contributing to this increase are modifiable by psychosocial treatments [e.g., obesity, quality of sleep; (53–56)]. Therefore, an innovative digital psychosocial treatment based on proven, data-driven strategies that patients can access at their convenience could create significant positive impact across multiple dimensions.

From a scientific and technical perspective, the results from this project will have a ground-breaking positive effect on how we think about and treat adolescents with chronic pain. That is, the project will help to improve our understanding of the needs of all the interested stakeholders (i.e., adolescents with chronic pain, their parents, healthcare professionals, and their teachers) in relation to the management of chronic pain. In addition, it will also provide valuable insights into how the digital treatment can be made more appealing for integration into mainstream healthcare procedures. Moreover, by evaluating DigiDOL-Ad in real-world settings, we will gather essential data on the digitally delivered psychosocial-based treatment to support adolescents in managing chronic pain. Currently, there is a significant lack of data on effective remotely delivered treatments for this group (57). This can deepen the understanding of chronic pain mechanisms and its management in adolescents, informing future therapeutic approaches. Digital treatments can reach adolescents in underserved or remote areas where access to specialized pain management services is limited. Therefore, DigiDOL-Ad, by reducing geographical and financial barriers, will also help promote equitable healthcare access. Although this initial phase focuses on feasibility and usability, our future randomized controlled trial (RCT) will enable us to quantify the intervention's broader impact, including potential healthcare cost savings and the number of adolescents who could benefit from this approach.

As described previously, we will develop a website for teachers with resources and information to assist students with chronic pain. Chronic pain can impact academic performance, attendance, and overall school functioning (8, 58, 59). Educators can play a crucial role in supporting students with chronic pain (60), yet they often lack the necessary resources and knowledge to effectively manage these students' unique needs (61–63). Developing a dedicated website with comprehensive resources and information can bridge this gap, offering numerous advantages for educators and students alike. Such a platform can help reduce absenteeism, improve communication between teachers, students, and their families, and provide guidance on appropriate attitudes and responses to chronic pain. By equipping educators with the right tools, we could help create a more supportive and understanding school environment for students with chronic pain.

The impact of this digital treatment is expected to be significant at both structural and economic levels. Socially, the study findings may prompt a shift in healthcare practices, reinforcing that chronic pain is not solely a physical condition and advocating for collective responsibility in health management.

Economically, chronic pain poses a substantial public health challenge with significant direct and indirect costs for families and society (64, 65). Implementing this digital treatment could thus reduce the economic burden of chronic pain on the healthcare system. For example, if found to be effective, widespread use and prescription of this digital treatment could significantly reduce the number of patients requiring hospital visits. Additional cost savings would arise from reduced direct expenses (e.g., fewer complementary tests, reduced hospital personnel time) and indirect expenses (e.g., fewer missed workdays for families). Furthermore, the economic impact of these outcomes may stimulate further research and the development of new therapeutic procedures, potentially generating new employment opportunities. Our current study primarily focuses on establishing the feasibility, usability and associated effects of using the digital treatment, a comprehensive economic evaluation will be undertaken in a subsequent phase. This future RCT will provide detailed figures on the direct and indirect costs of chronic pain and quantify the potential cost savings that could be achieved by implementing this digital treatment, thereby reducing the economic burden on the healthcare system.

### 3.2 Limitations

The key challenges anticipated in this project are related to (1) recruitment and inclusion of participants in the different studies, due to abandonment of a clinician/chronic pain program, and the difficulty to involve stakeholders in the FGs and usability studies; (2) privacy and data protection issues; (3) as a digital intervention, potential technical difficulties or poor usability could affect participants engagement; and (4) the content on companion websites for parents and teachers might be misunderstood, leading to unintended consequences. The mitigation actions (*contingency plan*) addressed to solve recruitment issues are as follows, depending on the causes: (a) monitoring constantly the involvement of clinicians, having a close and continued relationship with them (in relation to this, we have planned to send monthly updates to clinicians, and award a prize to the program providing the highest number of referrals), and (b) the Principal Investigator, in ongoing meetings with all stakeholders, will inform them on the project to raise interest and gain trust in the outcomes of the research proposal. In relation to (2) privacy data issues, we plan that the data collected during the implementation process will not leave the local secure networks where the data will be hosted at the university premises. Moreover, in relation to technical and usability issues, we have planned iterative usability testing phases and will provide dedicated technical support to identify and resolve any issues promptly. Finally, in relation to misinterpretation of information, the content will be developed in consultation with experts, and clear guidelines for interpretation and use will be provided.

Finally, another limitation of this project is the use of a single-group design to study the treatment. Without a control group for comparison, it is challenging to determine whether the observed outcomes are genuinely attributable to the treatment or influenced by external factors such as the placebo effect, natural progression of the condition, or other variables. If the evaluation study demonstrates positive results, a randomized controlled trial will be conducted in the future to validate and strengthen the findings.

## Author contributions

JM: Conceptualization, Funding acquisition, Methodology, Resources, Supervision, Writing – original draft, Writing – review & editing. AS: Project administration, Writing – review & editing. SM: Writing – review & editing. PI: Writing – review & editing. RW: Visualization, Writing – review & editing. CN: Writing – review & editing. MA: Writing – review & editing. JL: Writing – review & editing. EM: Writing – review & editing. PR: Writing – review & editing. AS: Writing – review & editing. VS: Writing – review & editing. AV: Writing – review & editing. Rd: Writing – review & editing. FR-B: Writing – review & editing.
