# Correction: Development, implementation and evaluation of a digital treatment for adolescents with chronic pain: a protocol for a multi-phase study

**DOI:** 10.3389/fdgth.2026.1868503

**Published:** 2026-06-16

**Authors:** Jordi Miró, Ariadna Sampietro, Sonia Monterde, Pablo Ingelmo, Rikard K. Wicksell, Carme Nolla, Mercedes Alonso, Juan José Lázaro, Ernesto Martínez, Paloma Rubio, Armando Sánchez, Vanessa Sánchez, Alvaro Vázquez, Rocío de la Vega, Francisco Reinoso-Barbero

**Affiliations:** 1 Unit for the Study and Treatment of Pain—ALGOS, Research Center for Behavior Assessment (CRAMC), Department of Psychology, Universitat Rovira I Virgili, Tarragona, Spain; 2 Institut D’Investigació Sanitària Pere Virgili, Universitat Rovira I Virgili, Tarragona, Spain; 3 Unit of Physical Therapy, Department of Medicine and Surgery, Faculty of Medicine and Health Sciences, Universitat Rovira I Virgili, Tarragona, Spain; 4 Department of Anesthesiology, Montreal Children’s Hospital, The Edwards Family Interdisciplinary Centre for Complex Pain, Montreal, QC, Canada; 5 Department of Anesthesia, McGill University, Montreal, QC, Canada; 6 Research Institute, Alan Edwards Centre for Research on Pain, McGill University Health Center, Montreal, QC, Canada; 7 Department of Clinical Neuroscience, Karolinska Institutet, Stockholm, Sweden; 8 Pain Clinic, Capio St Göran Hospital, Stockholm, Sweden; 9 Health Care Center Xarxa Tecla, Tarragona, Spain; 10 Pediatric Pain Unit, Pediatric Anesthesiology Department, La Paz University Hospital, Madrid, Spain; 11 Department of Anaesthesia and Pain Therap, Hospital Sant Joan de Déu, Barcelona, Spain; 12 Department of Anesthesia, Children University Hospital Niño Jesús, Madrid, Spain; 13 Pediatric Chronic Pain Unit, Anesthesia Department, 12 de Octubre Universitary Hospital, Madrid, Spain; 14 Pediatric Pain Unit, Pediatric Anaesthesiology Section, Critical Care and Pain Therapy Department, Miguel Servet University Hospital, Zaragoza, Spain; 15 Servei D’Anestesiologia, Reanimació I Tractament del Dolor, Hospital Infantil I Hospital de la Dona, Hospital Universitari Vall D’Hebron, Barcelona, Spain; 16 Servicio de Anestesiología y Reanimación, Unidad de Dolor, Hospital Universitari Son Espases, Palma, Spain; 17 Department of Personality, Evaluation, and Psychological Treatment, Faculty of Psychology and Speech Therapy, University of Málaga, Málaga, Spain; 18 Instituto de Investigación Biomédica de Málaga y Plataforma en Nanomedicina (IBIMA Plataforma BIONAND), Málaga, Spain

**Keywords:** adolescents, chronic pain, digital health, digital therapeutics, mobile application, pain management

The funder Spanish Ministry of Science, Innovation and Universities, FPU predoctoral fellowship FPU23/03355 to Ariadna Sampietro was erroneously omitted. And the funders MICIU/AEI/10.13039/501100011033 and ESF+, grant PREP2022-000864 to Ariadna Sampietro were erroneously omitted.

The corrected Funding statement appears below:

The author(s) declare that financial support was received for the research and/or publication of this article. This study is partially supported by a grant from the Spanish Ministry of Science and Innovation (ref. PID2022-142071OB-I00); and grants from the European Regional Development Fund (ERDF) and the Government of Catalonia (AGAUR; 2021SGR-730). JM’s work is supported by ICREA-Acadèmia. Ariadna Sampietro is supported by a FPU predoctoral fellowship from the Spanish Ministry of Science, Innovation and Universities (FPU23/03355), and was previously supported by grant PREP2022-000864 funded by MICIU/AEI/10.13039/501100011033 and by ESF+. RV’s work is supported by the Spanish Ministry of Science and Innovation with a Ramon y Cajal contract (RYC2018-024722-I). The Chair in Pediatric Pain is supported by Fundación Grünenthal, ANUBIS, Cosmetic group, and ESTEVE.

The original version of this article has been updated.

