# Supplementary material for: Development, implementation and evaluation of a digital treatment for adolescents with chronic pain: a protocol for a multi-phase study
Source: Front Digit Health. 2025 Jun 4;7:1555733. doi: 10.3389/fdgth.2025.1555733 (PMC12174134; doi:10.3389/fdgth.2025.1555733)
Supplement: Supplementary file 1 [file Supplementaryfile2.pdf]

Appendix 2. Consent forms.

UNIVERSITAT ROVIRA i VIRGILI

Full de consentiment informat

Títol de l'estudi: [1] Desenvolupament, Implementació i Efectivitat d'un tractament digital per a adolescents amb dolor crònic (DigiDOL-Ad); CEIPSA-2023-PRD-0033
Dades de contacte de l'investigador principal:[2] Jordi Miró Martínez; 977558179; jordi.miro@urv.cat; Dept de Psicologia, Carretera de Valls, S/N; Universitat Rovira i Virgili, Campus Sescelades, Edifici N0
Grup de recerca, si escau: ALGOS

Jo .... [3] amb DNI ....

- He llegit el full d'informació al participant sobre l'estudi del qual se m'ha entregat una còpia.
- He pogut fer preguntes i resoldre els meus dubtes sobre l'estudi i la meva participació.
- Comprenc la meva participació a l'estudi d'acord amb allò expressat al full d'informació al participant sobre l'estudi i de les respostes a les meves preguntes, així com els riscos i beneficis que comporta.
- Accepto que la meva participació és voluntària i dono lliurement la meva conformitat per participar a l'estudi.
- Conec que em puc retirar en qualsevol moment de la participació a l'estudi sense que això em pugui causar cap perjudici.
- Estic informat sobre el tractament que es realitzarà de les meves dades personals.
- Dono el meu consentiment per a l'accés i utilització de les meves dades en les condicions detallades al full d'informació al participant sobre l'estudi.

  ☐ **Sí** ☐**No**
- Un cop finalitzada la investigació, és possible que les dades obtingudes siguin d'interès per a altres estudis relacionats. En relació amb això, s'ofereixen les següents opcions:

  ☐ **NO autoritzar** l'ús de les seves dades en altres projectes d'investigació relacionats.

  ☐ **SÍ autoritzar** l'ús de les seves dades en altres projectes d'investigació relacionats.

[4] I per expressar aquest consentiment, el participant signa en data .... i lloc .... aquest full de consentiment:

[1] Del projecte de recerca, Tesi Doctoral, TFG o TFM. Si existeix, incloure també el codi o referència de l'estudi.
[2] Indicar les dades de contacte de l'investigador principal; nom, telèfon, correu electrònic i ubicació física.
[3] Indicar el nom i cognoms del participant.
[4] Si el participant pot prestar lliurement el consentiment utilitzar aquest redactat fins a la següent nota el redactat del qual es pot eliminar.

Signatura del participant ....

[5] I per expressar aquest consentiment, el representant legal del participant signa en data .... i lloc .... aquest full de consentiment:

Nom del representant legal..................................

Relació del representant legal amb el participant ....

Signatura del representant legal ....

---

[5] Si el participant no pot llegir o escriure, és menor de 14 anys o per qualsevol altre motiu no pot prestar lliurement el consentiment és necessari que el seu consentiment el presti el tutor o representant legal. En aquest cas utilitzarem aquest redactat i eliminarem el corresponent a la nota anterior.

Projectes de recerca / Tesis doctorals / Treballs de Fi de Màster / Treballs de Fi de Grau de la URV

Informació bàsica de protecció de dades

Informació bàsica sobre protecció de dades (format tabular)

INFORMACIÓ DE PROTECCIÓ DE DADES PERSONALS	
**Responsable**	El responsable del tractament de les seves dades personals és la Universitat Rovira i Virgili amb CIF Q9350003A i amb domicili fiscal al carrer de l'Escorxador, s/n, 43003 de Tarragona.
**Finalitat**	Participar en ....[6] en els termes que es descriuen al full d'informació al participant. En el cas que l'estudi prevegi la publicació, difusió i reutilització dels resultats obtinguts incloent dades personals, les dades personals seran utilitzades per a aquesta finalitat sempre que l'interessat hagi atorgat el seu consentiment.
**Drets**	Pot exercir els drets d'accés, rectificació, supressió, portabilitat, limitació o oposició al tractament, mitjançant un escrit adreçat al Registre General de la URV a la mateixa adreça del domicili fiscal o mitjançant la seva presentació al Registre General de la Universitat, presencialment o telemàtica, segons s'indica a https://seuelectronica.urv.cat/registre.html.
**Informació addicional**	Pot consultar informació addicional sobre aquest tractament de dades personals denominat......................[7] i els seus drets al Registre d'Activitats del Tractament de la URV publicat a https://seuelectronica.urv.cat/rgpd on també s'hi pot consultar la Política de Privacitat de la URV. Així mateix, pot consultar aquesta informació al Full d'informació al participant sobre l'estudi. Addicionalment, pot adreçar als nostres delegats de protecció de dades qualsevol consulta sobre protecció de dades personals a la direcció de correu electrònic del dpd@urv.cat.

[6] Indicar "el projecte de recerca" en el cas que es tracti d'un projecte de recerca, "l'estudi de la Tesis Doctoral" en el cas que es tracti d'una tesi doctoral o "l'estudi del Treball Final de Grau o de Màster" en el cas que es tracti d'un TFG o TGM.
[7] Indicar en cursiva *"Projectes de recerca de la URV"*, *"Treballs de Fi de Grau o de Màster de la URV"* o *"Tesis Doctorals de la URV"* segons correspongui
