# Supplementary material for: Development, implementation and evaluation of a digital treatment for adolescents with chronic pain: a protocol for a multi-phase study
Source: Front Digit Health. 2025 Jun 4;7:1555733. doi: 10.3389/fdgth.2025.1555733 (PMC12174134; doi:10.3389/fdgth.2025.1555733)
Supplement: Supplementary file 2 [file Supplementaryfile1.docx]

**Appendix 1. WHO Trial Registration Data Set.**

| Data category | Information |
| --- | --- |
| Primary Registry and Trial Identifying Number | ClinicalTrials.gov; Number: Waiting |
| Date of Registration in Primary Registry | 12/18/2024 |
| Source(s) of Monetary or Material Support | A grant from the Spanish Ministry of Science and Innovation (PID2022-142071OB-I00); and grants from the European Regional Development Fund (ERDF) and the Government of Catalonia (AGAUR; 2021SGR-730). |
| Scientific Title | Development, Implementation and Effectiveness of a Digital Treatment for Adolescents with Chronic Pain: Protocol for a multi-phase study. |
| Countries of Recruitment | Spain |
| Health Condition(s) or Problem(s) Studied | Chronic pain |
| Intervention(s) | Psychosocial digitally administered treatment for adolescents with chronic pain |
| Key Inclusion and Exclusion Criteria | Inclusion criteria: (1) 12-18 years old, (2) non-oncology secondary chronic pain problem, (3) internet access, (4) a parent willing to participate, and (5) informed consent/assent |
|  | Exclusion criteria: cognitive or language problems that interfere with assessment and treatment |
| Study Type | Type of study:   - Interventional |
|  | Study design:   - Allocation: non-randomized - Masking: N/A - Assignment: single arm - Primary purpose: treatment |
|  | Phase:   - N/A |
| **Date of First Enrollment** | 10/2025 |
| **Target sample Size** | 195 |
| **Recruitment Status** | Pending |
| **Primary Outcome(s)** | - Pain intensity, Numeric Rating Scale, before/after treatment and 3 months follow-up. - Pain interference, PROMIS-Pediatric Pain Interference, before/after treatment and 3 months follow-up. - Global impression of change, a question about the overall perception of change in a patient's condition after the treatment, after treatment and 3 months follow-up. |
| **Key Secondary Outcomes** | - Pain-related self-efficacy, Pain Self-Efficacy Questionnaire (PSEQ), before/after treatment and 3 months follow-up. - Concerns about pain, University of Washington Concerns About Pain (UW-CAP), before/after treatment and 3 months follow-up. - Pain attitudes, Pediatric version of the Survey of Pain Attitudes (Peds-SOPA), before/after treatment and 3 months follow-up. - Functional disability, Functional Disability Inventory (FDI), before/after treatment and 3 months follow-up. - Anxiety, PROMIS-Anxiety, before/after treatment and 3 months follow-up. - Depressive symptoms, PROMIS-Depression, before/after treatment and 3 months follow-up. - Sleep disturbance, PROMIS-Sleep disturbance, before/after treatment and 3 months follow-up. - Fatigue, Silhouettes Fatigue Scale (SFS), before/after treatment and 3 months follow-up - Treatment perceptions, questions about how easy to use and how helpful the taught skills are, after treatment and 3 months follow-up - Adherence, use of digital treatment, after treatment and 3 months follow-up |
